# The Need for Strengthening Health Information Dissemination Toward Indoor Residual Spraying for Malaria Prevention in Malarious Area of Ethiopia

**DOI:** 10.3389/fpubh.2022.913905

**Published:** 2022-06-13

**Authors:** Wubayehu Mekasha, Chala Daba, Asmamaw Malede, Sisay Abebe Debela, Mesfin Gebrehiwot

**Affiliations:** ^1^Angolela and Tera Woreda Health Office, Debre Birhan, Ethiopia; ^2^Department of Environmental Health, College of Medicine and Health Sciences, Wollo University, Dessie, Ethiopia; ^3^Department of Public Health, College of Medicine and Health Sciences, Salale University, Fiche, Ethiopia

**Keywords:** community acceptance level, Ethiopia, health interventions, indoor residual spraying, malaria

## Abstract

**Introduction:**

Malaria remains prevalent in developing countries. This is particularly true among the community who are prone but do not apply malaria prevention and controlling strategies. In one of the malarious areas of Ethiopia (Shewa Robit), the acceptance level of indoor residual spraying (IRS) is indicated to be low as per guidelines. However, factors determining communities' acceptance of IRS are not well-investigated. Hence, this study was designed to identify the determinants for the acceptance of IRS in order to indicate priorities for malaria prevention and control.

**Methods:**

A community-based cross-sectional study design was used among 649 households in Shewa Robit town, from February to March 2021. Households were selected from five IRS-targeted kebeles. Data were collected using structured questionnaire. A multivariable logistic regression model was used to identify the independent factors associated with the acceptance of IRS.

**Results:**

The response rate in this study was 98%. The proportion of community who accepted the IRS for malaria prevention was 56.5% [95% confidence interval (*CI*): 52.7–60.2%]. Being male [adjusted odds ratio (*AOR*) = 2.21, 95% *CI*: 1.32–3.72], having good knowledge (*AOR* = 2.25, 95% *CI*: 1.33–3.84), did not paint/re-plaster the wall after spraying (*AOR* = 3.99, 95% *CI*: 2.36–6.76), did not perceive any side effects after spraying (*AOR* = 1.82, 95% *CI*: 1.11–2.99), effectiveness of previous IRS (*AOR* = 2.99, 95% *CI*: 1.85–4.84), non-utilization of long-lasting insecticide-treated net (LLIN) (*AOR* = 0.52, 95% *CI*: 0.33–0.84), and spraying the house at the right season (*AOR*: 2.14, 95% *CI*: 1.11–4.13) were determinant factors for the acceptance of IRS.

**Conclusions:**

To increase the acceptance level of IRS among the communities, health interventions and services should focus on the awareness creation toward the effectiveness of IRS, proper spraying time/season, and side effects of IRS. Therefore, strengthening health information dissemination could help promote the acceptance of IRS.

## Introduction

Malaria continues to be a global public health problem (1). For example, in 2018, 405,000 deaths and 228 million cases were reported. Of these deaths, about 67% (272,000) were under-five children (2). Despite the implementation of malaria prevention and control strategies in Africa, malaria remains a public health challenge. According to the World Health Organization (WHO) report, around 213,000 malaria cases and 380,000 deaths were reported in Africa (2). Beyond morbidity and mortality, malaria causes huge economic loss (3).

Ethiopia is one of the African countries where malaria is endemic and 68% of its population are at risk of contracting malaria infection (4–6). There were more than 1.2 million malaria cases in 2018. Of this, 4,782 people were died (2). This is mainly associated with the topography and climate condition, which are more suitable for the reproduction of malaria vector (7). A retrospective study done in Ethiopia over 16 years period (2000–2016) also showed that the burden of malaria remains high and accounts over five million cases and thousands of deaths annually (8). For this reason, different intervention approaches such as indoor residual spraying (IRS), early diagnosis and prompt treatment, and insecticide-treated mosquito nets (ITNs) are being implemented to combat malaria. Other prevention and control strategies also include operational research and surveillance, environmental management, and monitoring and evaluation systems that provide appropriate information (9, 10). Among WHO's recommended malaria prevention and control strategies, IRS is considered as the most effective (11) as it acts against the mosquitoes' indoor biting and resting habits (12).

Assuming that the vectors are mainly indoor resting, IRS would be effective if more than 85% of the households/structures in the area are covered as per the guidelines (11). However, to implement IRS, the community acceptance is a key factor. Evidences from the various studies showed that the acceptance of IRS is as low as 29% (13, 14). The potential side effects of IRS and doubts about its effectiveness (15, 16), fears of increasing other insects (17), and some socio-economic status and prior IRS experience (18) were determinant factors for poor IRS acceptance.

Shewa Robit is one of the malarious areas in Ethiopia, where malaria infection is a significant public health problem. The prevalence of malaria in the study area was reported to be 13.9% with *plasmodium falciparum* and *vivax* being the dominant parasite species (19). As for most other parts of the country, *Anopheles arabiensis* is indicated to be the predominant vector (9). As a result, the local health offices are implementing IRS to combat malaria. However, there is no evidence about determinants of IRS acceptance, which could hind the proper planning for well-targeted interventions. Hence, this study was undertaken with the aim of identifying factors determining the communities' acceptance toward IRS in Shewa Robit town, North Eastern Ethiopia. The findings of this study could help in providing insights into possible intervention approaches.

## Methods

### Study Area

The study was conducted in Shewa Robit town which is located ~225 Km far from Addis Ababa (capital of Ethiopia). It is found at an elevation of about 1,280 m above the sea level. According to the 2019 health office report, the total population of the town administration was estimated to be about 47,468 of which 24,493 (51.6 %) were women and 22,974 (48.4 %) were men, with an average family size of 4.3. The town has one health center, one governmental hospital, four urban health posts, and five rural health posts. The yearly temperature in the area ranges from 30.7 to 31.9°C while the minimum temperature ranges from 12.98 to 15.45°C. The mean annual rainfall is about 84.64 mm with uneven and scarce occurrence.

### Study Design and Period

A community-based cross-sectional study was employed to assess the determinant factors of communities' acceptance toward IRS in Shewa Robit town, North Eastern Ethiopia from February to March 2021.

### Source and Study Population

The source population was all the households in IRS-targeted kebeles (*kebele is the lowest administrative unit in Ethiopia*) of Shewa Robit town; whereas all randomly selected households in the IRS-targeted kebeles of Shewa Robit town were the study population. The selected household heads/wives from the study population were the study units.

### Inclusion and Exclusion Criteria

All households in IRS-targeted kebeles in Shewa Robit town were included for this study; whereas study participants who had a serious medical condition during data collection period were excluded.

### Sample Size Determination

The sample size was computed using the STATCALC application of EPI-INFO version 7 software with the assumptions of 80% power, 95% confidence interval (*CI*), and *p* = 79.9%. The “*p*” is IRS acceptance in Uganda (18). Accordingly, the calculated sample size becomes 602. By adding a 10% of non-response rate, the final sample was 662.

### Sampling Technique and Procedure

**Figure 1:**
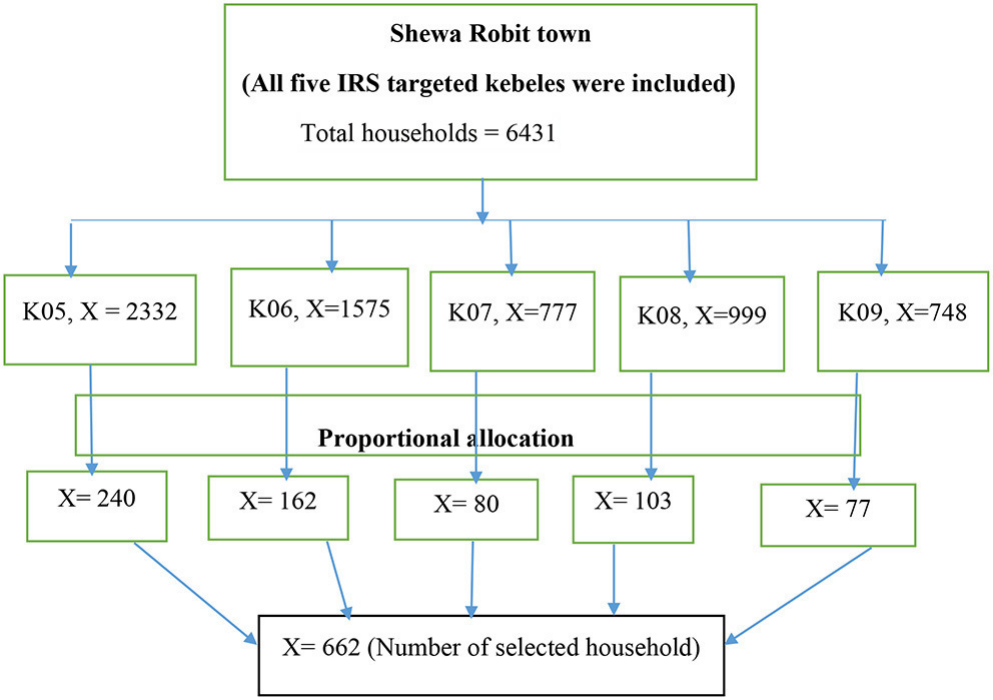
Sampling procedure. K = Kebele, X = Households, IRS = Indoor residual spraying.

The five IRS-targeted kebeles have a total of 6,431 households. The samples were proportionally allocated to each kebele based on their number of households (Figure 1). The households were selected through simple random sampling technique (lottery method) from the kebele registration book. Before the data collection, the sampling frame was designed by numbering the list of households using the registration book.

### Study Variables

The outcome variable in this study was acceptance of IRS (good or poor) and the independent variables were socio-demographic characteristic (sex, age, status of the household head, household size, occupation, educational status, and marital status), perception about IRS, knowledge about malaria, and practice of IRS (Appendix 1).

### Operational Definitions

In order to identify the acceptance of IRS (good/poor), the responses from nine IRS and malaria-related questions were computed. The correct answer for each item was given a score “1” and the incorrect answer was given a score “0.” Accordingly, a study participant who correctly answered above the mean score was considered as having a good acceptance level and *vice versa*. Similarly, in order to identify the knowledge of the community (good/poor) toward malaria, the responses from six knowledge questions were computed. The correct answer for each item was given a score “1” and the incorrect answer was given a score “0.” Accordingly, a study participant who correctly answered above the mean score was considered as having a good knowledge and *vice versa*.

### Data Collection Method

The data were collected using structured questionnaire which was adapted from the published articles (1, 13, 20–26). Data collection was conducted through face-to-face interview with the respondents. A household was revisited for two more times if the respondent (study participant) was not available during the first visit. The interviewers were recruited from the health centers based on their academic qualifications (two BSc Environmental health supervisors and four BSc nurses). Each participant gave their informed consent after explaining the aim of research and notifying them that their participation was completely voluntary.

### Data Quality Control

To ensure the quality of the data, the questionnaire was translated into the local language (Amharic). The pre-test was conducted on the 33 households out of the actual study area. In the same way, the interview and discussion guide were pre-tested for the quality. Necessary corrections were made on the questionnaire, interview guide, and discussion guide based on the results of the pre-test. The 2 days training was given to the data collectors and supervisors by the principal investigator on the objective of the study, methods of data collection, and ethical issues.

### Data Processing and Analysis

The collected data were checked, coded, and entered into EpiData 3.1 and analyzed by the SPSS version 25. Data cleaning and assumption checking were performed prior to the analysis. The descriptive statistical analysis was conducted to describe the characteristics of the study participants. The logistic regression model was fitted to identify the association between the independent variables and the dependent variable. Both bivariable (crude odds ratio) and multivariable (adjusted odds ratio) logistic regression analyses were computed with their 95% *CI*. From the bivariable analysis, the independent variables that show a strong association with the dependent variable at a *p*-value < 0.25 were included in the multivariable logistic regression model. In the multivariable logistic regression, the *p*-values < 0.05 were used to show statistical significance. Model fitness was checked using the Hosmer and Lemeshow test and multicollinearity was tested using collinearity diagnostic statistics (correlation matrix, variance inflation factor, and tolerance test).

## Results

### Socio-Demographic Characteristics

In this study, a total of 649 study participants have participated giving a response rate of 98%. The majority (413, 63.6%) of the study participants were above the age of 35 years. Regarding their occupational status, 520 (80.1%) of the study participants were farmers. Approximately 330 (50.8%) of the participants had no formal education (Table 1).

**Table 1:** Socio-demographic characteristics of the study population (*N* = 649) in Shewa Robit town, North Eastern Ethiopia, 2021.

**Variables**	**Categories**	**Frequency (%)**	**Acceptance level**	
			**Poor**	**Good**
Sex	Female	154 (23.7)	89	65
	Male	495 (76.3)	193	302
Age	≤ 35	236 (36.3)	124	112
	>35	413 (63.6)	158	255
Status of household head	Wife	105 (16.7)	67	38
	Husband	544 (83.8)	215	329
Marital status	Married	637 (98.1)	278	359
	Unmarried	12 (1.9)	4	8
Household size	1–5	150 (76.9)	57	93
	≥5	499 (23.1)	225	274
Occupation	Farmer	520 (80.1)	218	302
	Merchant	76 (11.7)	36	40
	Governmental employee	7 (1.1)	5	2
	Other self-business	46 (7.1)	23	23
Level of education	No formal education	330 (50.8)	129	201
	Elementary	195 (30)	105	90
	High school and above	124 (19.2)	48	76

### Knowledge of the Study Participants

Out of 649 respondents, 341 (52.5) respondents had good knowledge about malaria. In the present study, almost all (99.5%) study participants had information about malaria. Despite the fact that all study participants had information about malaria, only 293 (45.2%) study participants knew the breeding sites of mosquitoes. About three-fourths (72.3%) of the study participants knew that IRS can prevent malaria (Table 2).

**Table 2:** Knowledge of the study participants about malaria, North Eastern Ethiopia, 2021.

**Variable**	**Category**	**Frequency (%)**
Heard about malaria	No	3 (0.5)
	Yes	646 (99.5)
Know breeding site of mosquitoes	No	356 (54.8)
	Yes	293 (45.2)
Malaria is endemic in Shewa Robit	No	103 (15.9)
	Yes	546 (84.1)
Mode of transmission	Anopheles mosquitoes	454 (70%)
	Others	195 (30%)
IRS prevents malaria	No	180 (27.7)
	Yes	469 (72.3)
Malaria affects all age groups	No	210 (32.3)
	Yes	439 (67.6)
Level of knowledge	Good	341 (52.5)
	Poor	308 (47.5)

### Perception and Practice Toward IRS

In this study, 410 (63.2%) of the study participants believed that IRS is an effective malaria prevention and control strategy. Similarly, 350 (53.9%) of the study participants believed that they do not have water shortage for the IRS. Approximately 456 (70.2%) of the participants did not re-plaster/paint their house walls after spraying. Nearly two-thirds (64.7%) of the respondents received information about IRS prior to the spraying season (Table 3).

**Table 3:** Perception ad practice toward IRS among communities of Shewa Robit town, North Eastern Ethiopia, 2021.

**Variables**	**Category**	**Frequency (%)**	**Acceptance level**	
			**Good (%)**	**Poor (%)**
Received information before the spraying season	No	229 (35.3)	125	104
	Yes	420 (64.7)	157	263
Think the spraying season was the right time	No	86 (13.3)	50	36
	Yes	563 (86.7)	232	331
Effectiveness of IRS for malaria prevention and control	No	159 (32.6)	91	68
	Yes	410 (63.2)	104	306
Presence of adequate water for IRS	No	30 (4.6)	13	17
	Yes	619 (95.4)	269	350
Side effect	No	380 (58.6)	254	126
	Yes	269 (41.4)	121	148
Did not re-plaster or paint after spraying	No	193 (29.8)	114	79
	Yes	456 (70.2)	162	294
Use of long lasting insecticide net (LLIN)	No	341 (52.5)	204	137
	Yes	308 (47.5)	78	231

### IRS Acceptance Among the Study Participants

The proportion of the community that accepted IRS for malaria prevention is 56.5% (95% *CI*: 52.7–60.2%). Among all the study participants, 488 (75.2%) have had their houses sprayed during the previous spraying period. In this study, 76.4 and 90.1% of the study participants thought spraying were effective and perceived IRS as beneficial, respectively (Table 4).

**Table 4:** IRS acceptance among the study participants in Shewa Robit town, North Eastern Ethiopia, 2021.

**Variables**	**Category**	**Frequency (%)**	**Acceptance level**	
			**Poor (%)**	**Good (%)**
Sprayed the house in the previous round	No	161 (24.8)	127	34
	Yes	488 (75.2)	155	333
Bad smell of insecticide did not affect to spray the house	No	313 (48.2)	232	81
	Yes	336 (51.8)	50	286
Think spraying is beneficial	No	164 (25.3)	106	58
	Yes	485 (74.7)	176	309
Sprayers are trustful to enter into the house to spray	No	306 (47.1)	194	112
	Yes	343 (52.9)	88	255
Agree not to re-plaster and paint	No	297 (45.8)	208	89
	Yes	352 (54.2)	74	278
IRS is preferred method of malaria Prevention and control	No	232 (35.7)	202	30
	Yes	417 (64.3)	80	337
IRS reduces the nuisance of mosquitoes	No	60 (9.2)	47	13
	Yes	589 (90.8)	235	354
IRS reduces the chance of getting malaria	No	6 (0.9)	6	0
	Yes	643 (99.1)	276	367
Willingness to spray next season	No	225 (34.7)	196	29
	Yes	424 (65.3)	86	338
Acceptance level of IRS	Poor	282 (43.5%)
	Good	367 (56.5%)

### Reasons for IRS Refusal

Out of 649 respondents, 282 (43.4%) did not spray their houses for different reasons. The main reasons for not spraying their houses were side effects (95.4%), unpleasant odor/bad smell (88.3%), not aware of spraying season (81.2%), fear of food contamination (67%), and difficulty to move home furniture outside (42.9%) (Table 5).

**Table 5:** Reasons for IRS refusal (*N* = 282) in Shewa Robit town, North Eastern Ethiopia, 2021.

**Variables**	**Frequency**
Side effect	269 (95.4)
Unpleasant odor/ Bad smell	249 (88.3%)
Did not know spraying season	229 (81.2%)
Food contamination	189 (67%)
Difficulty to move home stuffs outside	121 (42.9%)

### Factors Associated With the Acceptance of IRS

In the multivariable logistic regression analysis, sex of study participants (being male), did not paint/re-plaster wall after spraying, good knowledge, absence of any side effect, effectiveness of the previous spraying, spraying season/time, and did not use long-lasting insecticide net (LLIN) showed significant association with the acceptance of IRS (Table 6). The likelihood of good IRS acceptance was two times higher among male study participants than others [adjusted odds ratio (*AOR*) = 2.21, 95% *CI*: 1.32–3.72]. Similarly, the study participants who had good knowledge were two times more likely to have good IRS acceptance than their counterparts (*AOR* = 2.25, 95% *CI*: 1.33–3.84). Once more, the odds ratio of good IRS acceptance was almost three times higher among study participants who previously had effective IRS than those with non-effective IRS. These households who did not paint/re-plaster after spraying within 3 months period were also 3.99 times more likely to have good acceptance of IRS than their counterparts (*AOR*: 3.99, 95% *CI*: 2.36–6.76) (Table 6).

**Table 6:** Multivariable analysis of factors associated with the acceptance of IRS in Shewa Robit, North Eastern Ethiopia, 2020.

**Variables**	**Category**	**Acceptance of IRS**		**COR (95% CI)**	**AOR (95% CI)**
		**Poor (%)**	**Good (%)**		
Sex	Female	89	65	1	1
	Male	193	302	2.14 (1.48–3.09)	2.21 (1.32–3.72)**
Age	≤ 35	124	112	1	1
	>35	158	255	1.79 (1.29–2.47)	1.35 (0.83–2.20)
Household size	>5	225	274	1	1
	1–5	57	93	0.75 (0.92–1.94)	0.89 (0.55–1.45)
Not painted/re-plastered after spraying	No	114	79	1	1
	Yes	162	294	6.88 (4.35–10.9)	3.99 (2.36–6.76)***
Level of knowledge	Poor	78	230	1	1
	Good	204	137	1.6 (0.16–0.62)	2.25 (1.33–3.84)*
Notice any side effects after spraying	Yes	121	148	1	1
	No	254	126	2.43 (1.62–3.64)	1.82 (1.11–2.99)*
Presence of adequate water for IRS	No	13	17	1	1
	Yes	269	350	1.61 (1.05–2.46)	1.74 (0.96–3.15)
Effectiveness of the previous spraying	No	91	68	1	1
	Yes	64	265	5.54 (3.65–8.40)	2.99 (1.85–4.84)***
Received information before spraying	No	125	104	1	1
	Yes	157	263	2.01 (1.45–2.79)	0.82 (0.46–1.45)
Utilization of LLIN	No	204	137	0.23 (0.16–0.62)	0.52 (0.33–0.84)*
	Yes	78	231	1	1
Perceive that the spraying season was the right time	No	50	36	1	1
	Yes	232	331	1.98 (1.25–3.14)	2.14 (1.11–4.13)*

1 = Reference group, *Significant at p-value <0.05, **p -value <0.01, ***p- value <0.001.

Furthermore, respondents who did not notice any side effects after spraying were 1.82 times more likely to have good IRS acceptance than others (*AOR*: 1.82, 95% *CI*: 1.11–2.99). Similarly, the odds ratio of good IRS acceptance was 52% higher among the study participants who did not use LLINs for malaria prevention as compared with study participants who have used LLINs (*AOR*: 0.52, 95% *CI*: 0.33–0.84) (Table 6).

## Discussion

This study was conducted with the aim of asessing determinants of community acceptance toward IRS in malarious area of Ethiopia. In this study, the magnitude of acceptance was 56.5% (95% *CI*: 52.7–60.2%). This magnitude of acceptance was lower than the findings of previous studies conducted in Nigeria (82.6%) (20), Uganda (79.9%) (18), Zambia (64%) (27), and Iran (94%) (1). On the other hand, our finding was higher than other similar studies done in Mozambique (41%) (13) and Assam (47.81%) (21). The variation in the level of IRS acceptance might be attributed to differences in the level of socio-demographic status, study setting, study design, measurement difference, and study period. The detailed discussion on the determinant factors is presented as follows.

Sex was identified as an important predictor of IRS acceptance. The odds ratio of good IRS acceptance was two times higher among male study participants as compared with female. This finding is consistent with other studies done in Uganda (25, 28). As men are usually the household heads (29), they receive detailed information related with health. Hence, they tend to accept and use malaria prevention strategies.

The study participants who had good knowledge were two times more likely to have good IRS acceptance than others. Similarly, IRS acceptance was higher among respondents who believed the previous spraying being effective. Although IRS is known to be effective in the control and elimination of malaria (30), in this study, approximately one-third of the study participants perceived that IRS is not effective. Similar findings were also reported in Mozambique (13) and north central Nigeria (20). This generally suggests that good knowledge and positive perception toward IRS enable the community to accept it.

The other statistically significant factor was perceived side effects of IRS. This finding revealed that the odds ratio of acceptance of IRS was lower among participants who perceived any side effects of IRS than their counterparts. Although more than two-thirds of the participants perceived IRS to be beneficial, the majority of respondents associated the benefits of IRS with the killing of mosquitos and other insects rather than the reduction of malaria disease. This concept contradicted with other studies done in Uganda (25). Our study, however, corroborates the findings of a study done in Iran (1), which indicated that respiratory disorders and headache, food contamination, not knowing the season of spraying, side effect, difficulty in furniture's movement, and unpleasant odor were the main reasons for IRS refusal. This signifies the need for extensive and regular health education programs.

The present study indicated that householders who did not paint/re-plaster their house wall after spraying tended to accept IRS. The odds ratio of good IRS acceptance was almost four times higher than among those respondents who did not paint/re-plaster after spraying as compared with others. This finding was in line with other studies which have been done in Eastern Ethiopia (31) and Lusaka, Zambia (24). This indicates that painting/re-plastering the internal walls (due to societal events, such as holy days or New Year) could reduce the effectiveness of IRS, and hence the acceptance of IRS.

Respondents who use LLINs were less likely to accept IRS as compared with study participants who did not use LLINs. This is consistent with the findings of studies done in Northwest Ethiopia (22) and Mozambique (23). This reveals that the preference for insecticide-treated nets is one of the main reasons for poor acceptance of IRS. However, WHO and Ethiopian federal ministry of health recommended the use of combinations of both bed net and IRS for effective malaria prevention (6, 32).

The current study indicated that individuals who perceived the spraying season as the right time had higher odds ratio of accepting IRS than their counterparts. Spraying their house at the right season could increase the performance of malaria prevention and control that can enhance the acceptance of IRS. Similar finding was also reported in Western Zambia (27).

## Conclusions

The acceptance of IRS for malaria prevention was only 56.5%. To increase the acceptance level of IRS among the communities, health interventions, and services should focus on awareness creation toward the effectiveness of IRS, proper spraying time/season, and side effects of IRS. Therefore, strengthening health information dissemination could help promote the acceptance of IRS.

## Data Availability Statement

The raw data supporting the conclusions of this article will be made available by the authors, without undue reservation.

## Ethics Statement

The studies involving human participants were reviewed and approved by the Wollo University research ethics review committee. Written informed consent for participation was not required for this study in accordance with the national legislation and the institutional requirements.

## Author Contributions

WM, CD, and MG were involved in data analysis and interpretation, did the preliminary assessment, and manuscript write up. SD and AM were involved in tool preparation, visualization, and methods. All authors contributed to the article and approved the submitted version.

## Conflict of Interest

The authors declare that the research was conducted in the absence of any commercial or financial relationships that could be construed as a potential conflict of interest.

## Publisher's Note

All claims expressed in this article are solely those of the authors and do not necessarily represent those of their affiliated organizations, or those of the publisher, the editors and the reviewers. Any product that may be evaluated in this article, or claim that may be made by its manufacturer, is not guaranteed or endorsed by the publisher.

## Abbreviations

AOR: adjusted odds ratio
CI: confidence interval
COR: crude odds ratio
IRS: indoor residual spraying
WHO: World Health Organization
ITN, insecticide-treated mosquito nets, and LLIN: long lasting insecticide net.
