# Supplementary material for: The Need for Strengthening Health Information Dissemination Toward Indoor Residual Spraying for Malaria Prevention in Malarious Area of Ethiopia
Source: Front Public Health. 2022 Jun 13;10:913905. doi: 10.3389/fpubh.2022.913905 (PMC9234660; doi:10.3389/fpubh.2022.913905)
Supplement: Supplementary file 1 [file Data_Sheet_1.pdf]

The need for strengthening health information dissemination towards indoor residual spraying for malaria prevention in malarious area of Ethiopia

Wubayehu Mekasha[1+], Chala Daba[2+], Asmamaw Malede[2], Sisay Abebe Debela[3], and Mesfin Gebrehiwot[2+]

[1]Angolela and Tera Woreda Health Office, Debre Birhan, Ethiopia

[2]Department of Environmental Health, College of Medicine and Health Sciences, Wollo University, Dessie, Ethiopia

[3]Department of Public Health, College of Medicine and Health Sciences, Selale University, Fiche, Ethiopia

+ Equally contributed to this work.

Correspondence: chaladaba293@gmail.com

Appendix 1 Questionnaire used to collect data about determinants of community acceptance of indoor residual spraying (IRS) for malaria prevention in Shewa Robit town, Northeastern Ethiopia

Questionnaire ID ___________

Name of the data collector ____________Signature__________Date ___/__/ 2021

Name of the supervisor _______________Signature ___________Date ___/__/2021

**Part 1: Socio-demographic characteristics of the study population (N=649) in Shewa Robit town, Northeastern Ethiopia, 2021**			
**S/no**	**Question**	**Response**	**Remark**
1.	Age?	__________years	
2.	Sex?	1=Female<br>2= Male	
3.	Status in household?	1= Wife<br>2= Husband	
4.	Household size?	1 = 1-5 years<br>2= >5 years	
5.	Marital status?	1= married<br>2= unmarried	
6.	Educational status?	1= No formal education<br>2= Elementary (1-8 grade)<br>3= High school (9-12	

		grade) and above	
7.	Occupation?	1= Farmer<br>2= Merchant<br>3= Government employee<br>4= others business	
**Part 2: Knowledge of the study participants about malaria, Northeastern Ethiopia, 2021**			
8.	Heard about malaria?	0= No<br>1= Yes	
9.	Know breeding site of mosquitoes?	0= No<br>1= Yes	
10.	Malaria is endemic in Shewa Robit?	0= No<br>1= Yes	
11.	Mode of transmission?	0= Anopheles mosquito<br>1= Others	
12.	IRS prevents malaria?	0= No<br>1= Yes	
13.	Malaria affects all age groups?	0= No<br>1= Yes	
**Part 3: Perception ad practice towards of IRS among communities of Shewa Robit town, Northeastern Ethiopia, 2021**			
14.	Received information before the spraying season?	0= No<br>1= Yes	
15.	Think the spraying season was the right time?	0= No<br>1= Yes	
16.	Effectiveness of IRS for malaria prevention and control?	0= No<br>1= Yes	

17.	Presence of adequate water for IRS?	0= No<br>1= Yes	
18.	Side effect?	0= No<br>1= Yes	
19.	Did not re-plaster or paint after spraying?	0= No<br>1= Yes	
20.	Use of Long Lasting Insecticide treated Nets (LLINs)?	0= No<br>1= Yes	
**Part 4: IRS acceptance among the study participants in Shewa Robit town, Northeastern Ethiopia, 2021**			
21.	Sprayed the house in the previous round?	0= No<br>1= Yes	
22.	Bad smell of insecticide did not affect to spray the house?	0=No<br>1= Yes	
23.	Think spraying is beneficial?	0= No<br>1= Yes	
24.	Sprayers are trustful to inter into the house to spray?	0=No<br>1=Yes	
25.	Agree not to re-plaster and paint?	0= No<br>1= Yes	
26.	IRS is preferred method of malaria prevention and control?	0= No<br>1=Yes	
27.	IRS reduces the nuisance of mosquitoes?	0= No<br>1=Yes	
28.	IRS reduces the chance of getting malaria?	0= No<br>1=Yes	

29.	Willingness to spray next season?	0= No<br>1=Yes	
**Part 5: Reason for IRS refusal (N=282) in Shewa Robit town, Northeastern Ethiopia, 2021 (more than one possible answers)**			
30.	Unpleasant odors/ Bad smell?		
31.	Difficult to take the furniture outside?		
32.	Food contamination?		
33.	Did not know spraying season?		
34.	Side effect		
